# Expanding Access to the Intrauterine Device in Public Health Facilities in Ethiopia: A Mixed-Methods Study

**DOI:** 10.9745/GHSP-D-15-00365

**Published:** 2016-03-25

**Authors:** Yewondwossen Tilahun, Sarah Mehta, Habtamu Zerihun, Candace Lew, Mohamad I Brooks, Tariku Nigatu, Kidest Lulu Hagos, Mengistu Asnake, Adeba Tasissa, Seid Ali, Ketsela Desalegn, Girmay Adane

**Affiliations:** a Pathfinder Ethiopia, Addis Ababa, Ethiopia; b Pathfinder International, Watertown, MA, USA; c Pathfinder International, Washington, DC, USA

## Abstract

In Ethiopia, modern contraceptive prevalence among currently married women nearly tripled over the last decade, but the method mix remains skewed toward short-acting methods. Since 2011, the Integrated Family Health Program (IFHP+), jointly implemented by Pathfinder International and John Snow Inc., has supported the Federal Ministry of Health to introduce intrauterine devices (IUDs) in more than 800 health centers across 4 regions to improve access to a wider range of methods. Between March and August 2014, Pathfinder conducted a mixed-methods study in 40 purposively selected health centers to assess shifts in the contraceptive method mix following introduction of IUDs using data from family planning registers; determine the characteristics of IUD users through a cross-sectional survey of 2,943 family planning clients who accepted the IUD; explore reasons for method discontinuation among 165 clients seeking IUD removal services; and identify facilitators and barriers to IUD use through focus group discussions (N = 115 clients) and key informant interviews (N = 36 providers, facility heads, and health office heads). Introduction of IUDs into the 40 health centers participating in the study was correlated with a statistically significant increase in the contribution of all long-acting reversible contraceptives (LARCs)—both IUDs and implants—to the method mix, from 6.9% in 2011 to 20.5% in 2014 (*P*<.001). Our study found that latent demand for the IUD was more prevalent than anticipated and that the method was acceptable to a broad cross-section of women. Of the 2,943 women who sought IUDs during the 6-month study period, 18.0% were new contraceptive users (i.e., those using a contraceptive method for the first time ever), 44.7% reported no educational attainment, 62.5% were from rural areas, and 59.3% were younger than 30 years old, with almost 3 in 10 (27.7%) under the age of 25. The most commonly cited reason for seeking IUD removal services was a desire to become pregnant (43% of women). Qualitative data indicated that while acceptability of the method is growing, limited community awareness, myths and misconceptions about the IUD, and infrastructure deficits at health centers must be addressed to further expand access to a broad range of contraceptive methods.

## Abstract (precis)

Following the introduction of IUDs into the Ethiopian public health sector, use of the method increased from <1% in 2011 to 6% in 2014 in a sample of 40 health facilities. This shift occurred in the context of wide method choice, following provider training, provision of post-training supplies, and community-based awareness creation. The IUD was acceptable to a diverse range of clients, including new contraceptive users, those with little to no education, those from rural areas, and younger women, thus suggesting a strong latent demand for IUDs in Ethiopia.

## INTRODUCTION

In sub-Saharan Africa, approximately 1 in 4 women has unmet need for contraception.^1^ Making a wide range of effective contraceptive methods available is critically important for satisfying this unmet need, for ensuring that women and couples have access to their method of choice, and for improving service quality.^2^ However, the contraceptive method mix in sub-Saharan Africa is skewed toward short-acting methods (which constitute a full 82% of modern contraceptive use in the region), while permanent methods and long-acting and reversible contraceptives (LARCs)—comprising implants and intrauterine devices (IUDs)—remain underutilized.^1^

In recent years, governments and partner organizations in the region (most notably in Ethiopia, Malawi, Rwanda, and Tanzania) have made a concerted effort to increase access to contraceptive implants (including Jadelle, Implanon, and Sino-implant II), driven by policy commitment, dedicated manufacturers, product price reductions, and successful provider training and task shifting.^3^^,^^4^ In these 4 countries, implant use has more than doubled over the last decade as women are increasingly drawn to the implant’s high effectiveness, long-acting nature, and discreet method of insertion (i.e., placed under the skin of the upper arm).^3^

Despite effectiveness levels similar to that of the implant, the IUD has received less attention in sub-Saharan Africa. This is due in part to limited provider training but also to perceived lack of demand given the mode of provision (requiring a pelvic examination and insertion of the device directly into the uterus), which could potentially deter clients in more conservative settings.^5^ However, recent initiatives to increase availability of IUDs in the region suggest that latent demand for this method may be more prevalent than previously thought.^5^ For example, the Supporting Access to Family Planning and Post-Abortion Care project, which increased access to IUDs in crisis-affected areas in Chad, the Democratic Republic of the Congo (DRC), and Pakistan, noted a subsequent upward trend of IUD use.^6^ Moreover, introduction of IUDs into the private health sector across 13 countries (including 6 located in sub-Saharan Africa) demonstrated the method’s acceptability to both women and adolescents.^5^ Despite this early evidence, the IUD remains largely underutilized in sub-Saharan Africa—especially in the public sector—and use has declined throughout the region from 6% in 2003 (roughly 1.2 million users) to 3% in 2012 (approximately 1.08 million users).^7^

The IUD has received little attention in sub-Saharan Africa due in part to limited provider training and perceived lack of demand among women.

### Ethiopian Context

Ethiopia has witnessed a significant increase in modern contraceptive prevalence among currently married women in the last decade, from 13.9% in 2005 to 27.3% in 2011 and 40.4% in 2014.^8^^-^^10^ Despite this substantial jump, the method mix remains skewed toward short-acting methods, as is the case in sub-Saharan Africa overall, with 84.7% of modern method users relying on pills, injectables, and male condoms for ongoing contraception.^10^ LARCs account for 14.6% of total modern contraceptive use among currently married women (12.1% for implants and 2.5% for IUDs), and permanent methods constitute 0.2% of total modern method use.^10^

The method mix in Ethiopia is skewed toward short-acting methods.

Reflecting the government’s commitment to expanding availability of implants and mirroring the broader regional trend throughout sub-Saharan Africa, the proportion of married women using implants has increased steadily over the last decade, from 0.2% in 2005 to 3.4% in 2011 and 4.9% in 2014.^8^^-^^10^ Use of IUDs, on the other hand, stagnated from 2005 to 2011 (0.2% and 0.3%, respectively), and despite a small increase to 1% in 2014, remains low.^8^^-^^10^ Knowledge of the IUD also lags, with just 38.9% of Ethiopian women reporting IUD knowledge, in comparison with 73.5% who report familiarity with implants.^10^

Recognizing the need to further expand the range of methods available in the public sector (through which 87% of women obtain contraception),^10^ the Ethiopian Federal Ministry of Health, in collaboration with partners supporting contraceptive services in the country, launched the “Ethiopia Intrauterine Contraceptive Device Scale-up Initiative (2011–2013)” in July 2010 (which has since been extended to 2017). This initiative aimed to increase access to and demand for the copper IUD—a non-hormonal, highly effective contraceptive method that can be used for up to 12 years.^11^ Prior to the government’s initiative, IUDs were available only through private NGO clinics located in larger, urban areas.

The Ethiopian government, with support from partners, launched an initiative in 2010 to expand access to IUDs and the range of methods available.

In December 2011, the Integrated Family Health Program (IFHP+), funded by the United States Agency for International Development (USAID)-supported Evidence to Action Project (E2A) and jointly implemented by Pathfinder International and John Snow Inc., began supporting the government’s IUD initiative in public-sector health centers in its 4 program regions: Amhara, Oromia, the Southern Nations, Nationalities, and People’s Region (SNNPR), and Tigray. IFHP+’s catchment area covers 300 districts and 60% of the population of these 4 regions. In this article, we describe IFHP+’s experience supporting the government to introduce IUDs into public-sector health centers in these 4 regions, subsequent shifts in the contraceptive method mix, characteristics of clients adopting IUDs, and reasons for method discontinuation.

## INTRODUCTION OF IUDS INTO PUBLIC-SECTOR HEALTH FACILITIES—THE LEARNING PHASE

From December 2011 to March 2012, IFHP+ conducted a “learning phase” that aimed to:

- Train a subset of clinical family planning providers on LARCs, including the IUD
- Initiate service provision
- Increase awareness of the IUD
- Inform future scale-up efforts

The learning phase was carried out in 128 health centers (32 per region) and their surrounding catchment areas. *Woreda* (district) health office heads selected these 128 health centers based on provision of contraceptive services; availability of at least 2 family planning providers to be trained; and availability of a supportive facility head or service provider to oversee targeted data collection. IFHP+ worked with its government counterparts to train a total of 256 providers (2 family planning providers per health center) on IUD insertion and removal, as well as all other contraceptive methods, during the learning phase. These competency-based trainings were composed of a 7-day theoretical and simulated practice component, followed by a 7-day clinical practicum at a high-volume facility.

To increase the likelihood of having sufficient client numbers for each training practicum and also to reach women with unmet need for contraception at the community level, IFHP+ worked with the government to engage health extension workers—a formalized cadre of frontline health workers in Ethiopia—in informing community members about the availability of all contraceptive methods, including IUDs, during practicums. These health extension workers incorporated sensitization on IUDs into the counseling they already provided at the community level and worked to dispel myths and misconceptions about the IUD among women, their partners, and communities. Health extension workers also worked with members of Ethiopia’s health development army—comprising individuals who promote health services within communities—to generate demand and convey information about IUDs at the community level. In addition, IFHP+ used vans with speakers to broadcast information about the availability of all contraceptive methods during upcoming trainings at marketplaces and other high-traffic community events. Lastly, the Federal Ministry of Health disseminated messages about IUDs through media channels (television and radio) during the scale-up initiative.

Providers counseled clients who sought services during the practicum on all short- and long-acting methods, screened them for eligibility as guided by the World Health Organization (WHO) “Medical Eligibility Criteria for Contraceptive Use” (4th edition), and provided clients with the method of their choice, including IUDs. At the end of these training sessions, IFHP+ provided post-training equipment and supplies (i.e., IUD kits and consumables) to each health facility involved in the learning phase. In this way, providers were able to immediately begin offering services at their respective health centers.

Given the sparse number of women requesting removal services during the learning phase (since IUDs were available only through private NGO clinics prior to the national scale-up initiative), IFHP+ trained providers on IUD removal using simulated models. In addition, trainees observed IUD removals among the very small number of clients who had received an IUD in NGO clinics and had sought removal services during the practical session of the learning phase.

Following the training, providers integrated IUDs into the range of services offered at their respective health centers, and health extension workers continued to create awareness of IUD service availability both at the community level during household visits and at the health post level (i.e., the lowest level of primary health care—one level below the health center).

**Figure:**
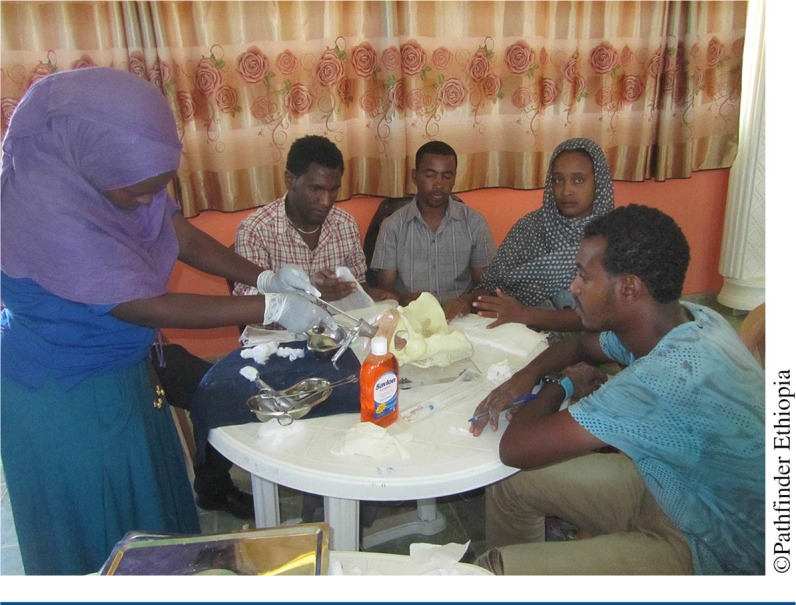
An instructor in Ethiopia demonstrates how to insert an IUD using a pelvic model.

At least once per month, skilled providers and Pathfinder staff provided post-training follow-up and mentorship at all health centers included in the learning phase. During these visits, the mentors used a checklist to assess adequate space, equipment, infection prevention measures, and client privacy during IUD insertion and removal. Mentors also assessed the quality of provider counseling and clinical skills as well as any challenges faced during service provision; recommendations from mentors were shared with district and regional health authorities. Three months following the training, one performance review meeting was convened by each of the 4 Regional Health Bureaus (i.e., Amhara, Oromia, SNNPR, and Tigray) to assess the achievements and challenges of the learning phase, with trainees, facility heads of the participating health centers, *woreda* health managers, family planning experts, and Pathfinder staff.

### Results of the Learning Phase

At the end of the learning phase (March 2012), assessments revealed that all 128 health centers routinely offered IUD insertion and removal services, and all were able to initiate the service immediately after the training. A total of 3,108 women in the 4 IFHP+ regions sought any contraceptive method at the 128 health centers during the learning phase. Among them, 992 (31.9%) accepted IUDs, of whom a remarkable 867 (87.4%) were new family planning acceptors (i.e., using contraception for the first time ever).

Following the successful learning phase, IFHP+ transitioned to the scale-up phase (beginning in April 2012), which aimed to capacitate all health centers and hospitals in the IFHP+ catchment area to offer a broadened method mix, including IUDs. Using the same training approach as was used during the learning phase, IFHP+ extended services to an additional 738 health facilities between April 2012 and September 2015; the scale-up phase continues to date. During both the learning and the scale-up phase, IFHP+ supported the distribution of IUDs to the health facilities through government channels.

## METHODS

To better understand client characteristics and inform future programming, we conducted a study from March through August 2014 (2 years into the scale-up phase) to assess the impact of IFHP+’s support for the Ethiopian government’s IUD initiative in program-supported regions (Figure). Our study had 3 specific objectives:

- Assess shifts in the contraceptive method mix following introduction of IUDs
- Identify the characteristics of clients choosing IUDs and describe the reasons for IUD discontinuation
- Identify facilitators and barriers to IUD use

**FIGURE:**
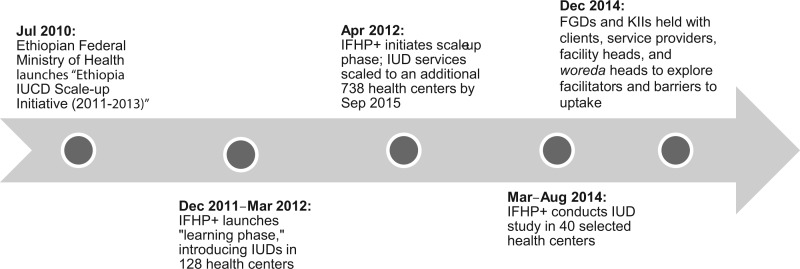
Timeline of Ethiopia's IUD Initiative, IFHP+’s Programmatic Support, and Pathfinder International’s Study Abbreviations: FGD, focus group discussion; IUD, intrauterine device; IFHP+, Integrated Family Health Program; KII, key informant interview.

We conducted a mixed-methods study composed of 3 specific components: (1) review of family planning data from health facility registers; (2) cross-sectional IUD client surveys; and (3) qualitative interviews with IUD clients and key informants.

### Health Facility Register Review

To assess shifts in the contraceptive method mix following introduction of IUDs, we retrospectively extracted and reviewed data from family planning registers in 40 purposively selected health centers supported by IFHP+ (10 from each program region). Health centers were selected based on: (1) high client load; (2) continued availability of IUD services (along with other contraceptive methods) since 2012; (3) willingness of the facility head and providers to collect data; and (4) accessibility of the facility for follow-up during the data collection period. We compared the contraceptive method mix among family planning clients during the 6-month study period (March–August 2014) with the 6-month period preceding initiation of the IFHP+ learning phase (July–December 2011). Contraceptive service delivery data from the 40 facilities indicate there are no significant seasonal differences in contraceptive uptake; thus, using data from 2 different 6-month periods should not have introduced bias. Methods included in the method mix and assessed were implants, IUDs, injectables, pills, and condoms. Lactational amenorrhea, withdrawal, and the Standard Days Method are not registered in health centers’ family planning service provision data, and permanent methods (i.e., vasectomy and tubal ligation) are not provided at the health center level in Ethiopia. Data from family planning registers were entered into an Excel document for data cleaning and management, and we performed chi-square pre-post statistical analyses using Epi-Info version 3.5.1.

### Cross-Sectional Client Surveys

To complement the family planning register review, we conducted a cross-sectional survey of family planning clients to better understand the characteristics of IUD acceptors. All women seeking IUD insertion services at these 40 health centers during the 6-month study period (March–August 2014) were eligible to participate in our survey. Health service providers counseled women on all contraceptive methods available, and if a woman expressed interest in the IUD, the provider informed her of the study and asked her if she would like to participate. All clients were informed that they would still be eligible to receive the IUD even if they refused to participate in the study. Providers used a consent form translated into local languages to obtain consent, and participants provided written consent. Providers then used a pretested, semi-structured questionnaire to collect sociodemographic and family planning information from all clients seeking IUD insertion who consented to participate.

The sample size for IUD acceptors was calculated using single population proportion formulas for each region where the IUD scale-up had been conducted. Since no previous studies had been conducted to determine the characteristics of IUD users, we assumed a population proportion of 50% of females aged 15–49 to maximize the sample size. Taking a 5% margin of error, 95% confidence level, and a design effect of 2, the sample size was calculated at 768 IUD clients per region. Adding 5% for non-response, the required sample size was 806 per region, resulting in a total sample size of 3,224 IUD service users across the 4 regions. During the 6-month study period, a total of 2,943 clients accepted the IUD, and all these clients agreed to participate in the study (for a response rate of 100%). Thus, we were able to reach 91% of the required sample size (2,943/3,224) during the study period.

We also conducted a separate cross-sectional survey among women seeking IUD removal services at these 40 health centers during the 6-month study period to understand client characteristics and to describe the reasons for IUD discontinuation. All women seeking IUD removal services at the 40 health centers were eligible to participate. Clients were informed that they would still be eligible for IUD removal even if they refused to participate in the study. Providers used a consent form translated into local languages to obtain consent, and participants provided written consent. Providers then used a pretested, semi-structured questionnaire to collect sociodemographic and family planning information from all clients seeking IUD removal who consented to participate. Given that very few clinics offered IUD services prior to 2011, our sample size for the discontinuation cross-sectional survey was limited; we enrolled a total of 165 clients. For both the IUD acceptor and the IUD discontinuation data sets, we derived basic descriptive statistics using SPSS version 20.

### Qualitative Interviews

To explore facilitating factors and barriers to IUD use, we conducted a series of focus group discussions (FGDs) and key informant interviews (KIIs) at 12 of the 40 health facilities participating in the study. These 12 facilities were purposively selected based on client volume during the 6-month period, with 1 high-, 1 medium-, and 1 low-volume facility selected from each of the 4 regions. We used semi-structured interview guides to facilitate 1 FGD at each facility with a total of 115 participants who had sought either IUD insertion or removal services. KIIs were held with 36 stakeholders from the 12 facilities (i.e., 1 service provider, 1 health facility head, and the *woreda* health office head associated with each health center). All interviews were conducted in the local language and were recorded, transcribed, and finally translated into English. A bilingual member of our team confirmed the accuracy of the English translation. Qualitative interviews were analyzed using content analysis to identify key recurring themes and to explore the nature of divergent views.

### Ethics Review

Ethical clearance for this study was obtained from the ethical review committee within each of the Federal Ministry of Health Regional Health Bureaus in Amhara, Oromia, SNNPR, and Tigray. All survey participants provided written informed consent before the interviews were conducted, and all data collection and analysis were conducted according to international principles of maintaining privacy and confidentiality of personal information.

## RESULTS

### Shifts in the Contraceptive Method Mix Following Introduction of IUDs

Table 1 shows the contraceptive method mix in the 40 health centers during the 6 months immediately preceding IFHP+ support for the government’s IUD initiative (July–December 2011) compared with the method mix during the 6-month study period (March–August 2014). The proportion of IUD users increased 14-fold, from 0.4% of total users in 2011 to 5.7% in 2014. Moreover, the overall contribution of LARCs to the total method mix increased substantially from 6.9% to 20.5%, with implant use doubling from 6.6% to 14.8%. The overall proportion of users relying on short-acting methods declined from 93.1% to 79.5%, with injectable use declining by 11.5 percentage points (from 75.8% to 64.3%) and oral contraceptive use declining by 5.9 percentage points (from 13.2% to 7.3%). The proportion of clients relying on condoms, however, nearly doubled from 4.1% to 7.9%.

The proportion of IUD users among family planning clients increased 14-fold in the study area, from <1% in 2011 to almost 6% in 2014.

**TABLE 1:** Contraceptive Method Mix among Family Planning Clients in 40 Selected Ethiopian Health Centers Before (Jul–Dec 2011) and After (Mar–Aug 2014) the IUD Scale-Up Initiative

Contraceptive Method	Before No. (%)	After No. (%)	Percentage Point Difference	*P* Value
**Long-acting reversible methods**	3,436 (6.9)	10,529 (20.5)	13.6	<.001
Implants	3,260 (6.6)	7,586 (14.8)	8.2	<.001
IUDs	176 (0.4)	2,943 (5.7)	5.3	<.001
**Short-acting methods**	**46,242 (93.1)**	**40,713 (79.5)**	**-13.6**	**<.001**
Injectables	37,647 (75.8)	32,934 (64.3)	-11.5	<.001
Pills	6,551 (13.2)	3,733 (7.3)	-5.9	<.001
Condoms	2,044 (4.1)	4,046 (7.9)	3.8	<.001
**Total**	**49,678 (100.0)**	**51,242 (100.0)**		

### Characteristics of Clients Choosing IUDs

During the 6-month study period, a total of 2,943 clients accepted IUDs at the 40 health centers included in the study. As Table 2 demonstrates, most IUD clients (62.5%) resided in rural areas, the vast majority (92.0%) were married, and just under half (44.7%) reported no educational attainment. The age distribution of users was relatively balanced among women aged 20 and older (about 20% in each 5-year age group), with the exception of a slightly higher proportion (31.6%) of women aged 25–29 accepting the IUD. While most IUD acceptors were aged 20 and older, a substantial 6.2% were younger than 20. Many acceptors had previously been pregnant 2–4 times (48.5%), most had given birth 2–4 times (52.8%), and most had 2–4 living children (53.8%). Still, those who had never been pregnant comprised a notable 7.5% of users. The majority of IUD clients (80.8%) heard about the service from a health care provider stationed at a health center (i.e., nurses, midwifes, or health officers), while 17.1% were informed about the availability of IUD services by a health extension worker.

**TABLE 2:** Background Characteristics of IUD Users (N = 2,943)

	No. (%)		No. (%)
Region		No. of previous pregnancies	
Oromia	926 (31.5)	0	221 (7.5)
Tigray	729 (24.8)	1	484 (16.4)
Amhara	657 (22.3)	2–4	1,426 (48.5)
SNNPR	631 (21.4)	>4	812 (27.6)
Residence		Mean (SD)	3.3 (2.3)
Rural	1,838 (62.5)	No. of births^a^	
Urban	1,104 (37.5)	0	38 (1.4)
Missing	1 (0.0)	1	477 (17.5)
Marital Status		2–4	1,436 (52.8)
Married/living together	2,709 (92.0)	>4	759 (27.9)
Never married	190 (6.5)	Missing	12 (0.4)
Divorced/separated/widowed	42 (1.4)	Mean (SD)	3.5 (2.1)
Missing	2 (0.1)	No. of living children^b^	
Religion		0	12 (0.4)
Orthodox	1,929 (65.5)	1	500 (18.6)
Protestant	540 (18.3)	2–4	1,444 (53.8)
Muslim	393 (13.4)	>4	715 (26.6)
Catholic	74 (2.5)	Missing	13 (0.5)
Traditional	3 (0.1)	Mean (SD)	3.4 (2.0)
Missing	4 (0.1)	Sources of information about IUD service	
Educational level		Health professional^c^	2,377 (80.8)
No education	1,316 (44.7)	Health extension worker	502 (17.1)
Primary	1,114 (37.9)	Media	35 (1.2)
Secondary	356 (12.1)	Health development army	11 (0.4)
More than secondary	152 (5.2)	Relatives	5 (0.2)
Missing	5 (0.2)	Mobile van	3 (0.1)
Age		Others^d^	10 (0.3)
<20	182 (6.2)		
20–24	633 (21.5)		
25–29	930 (31.6)		
30–34	614 (20.9)		
>34	580 (19.7)		
Missing	4 (0.1)		
Mean (SD)	28.0 (6.0)		

Abbreviations: IUD, intrauterine device; SD, standard deviation; SNNPR, Southern Nations, Nationalities, and People’s Region.

a Denominator is 2,722 women who had previously been pregnant.

b Denominator is 2,684 women who had ever given birth.

c Includes nurses, midwives, and health officers working in health centers.

d Includes neighbors and school.

Table 3 shows ever use of family planning and the method used (if any) at the index visit for the 2,943 women choosing the IUD during the 6-month study period. Nearly 1 in 5 (18.0%) IUD acceptors had never used a contraceptive method previously. Of the 81.3% of women who had used contraception in the past, 74.3% were shifting from a short-acting method—mostly from injectables (67.2%)—which corresponds with the overarching shift in the method mix from short-acting methods toward LARCs following introduction of IUDs (Table 1). A notable 14.8% of IUD acceptors who had previously used contraception were shifting from a contraceptive implant.

18% of IUD acceptors had never used a contraceptive method in the past.

**TABLE 3:** Previous Use of Contraception Among New IUD Acceptors

	No. (%)
**Ever used family planning**	**2,943 (100.0)**
Yes (Method shift)	2,394 (81.3)
No (New acceptors)	531 (18.0)
Missing	18 (0.6)
**Method used at index visit among those who had ever used family planning**	**2,394 (100.0)**
None^a^	258 (10.8)
Long-acting reversible methods	355 (14.8)
Implanon	292 (12.2)
Jadelle	51 (2.1)
Norplant	12 (0.5)
Short-acting methods	1,780 (74.3)
Injectables	1,609 (67.2)
Combined oral contraceptives	116 (4.8)
Condoms	7 (0.3)
Breastfeeding	11 (0.5)
Natural methods (withdrawal, SDM, etc.)	37 (1.5)
Missing	1 (0.0)

Abbreviations: IUD, intrauterine device; SDM, Standard Days Method.

a Had used family planning in the past but were not using any method at the time of their index visit.

### Reasons for IUD Discontinuation

Table 4 delineates the sociodemographic characteristics of the 165 clients seeking IUD removal services at the 40 health centers during the 6-month study period. The typical client seeking removal services was married (84.8%), aged 25 or older (69.7%), and had 2–4 living children (55.4%). Most clients reported either no educational attainment (37%) or primary school education (28.5%). Seven in 10 women (69.7%) seeking removal services during the study period had continued use for more than 1 year. Most of those seeking removal services (61.8%), however, reported using the IUD for 36 months or less.

**TABLE 4:** Characteristics of Women Seeking IUD Removal Services (N=165)

	No. (%)		No. (%)
Region		No. of previous pregnancies	
Oromia	63 (38.2)	0	23 (13.9)
Tigray	30 (27.9)	1	36 (21.8)
Amhara	46 (18.2)	2–4	71 (43.0)
SNNPR	26 (15.8)	>4	27 (16.4)
Residence		Missing	8 (4.8)
Rural	87 (52.7)	Mean (SD)	2.6 (2.3)
Urban	78 (47.3)	Ever given birth^a^	
Marital status		Yes	130 (91.5)
Married/living together	140 (84.8)	No	5 (3.5)
Never married	10 (6.1)	Missing	7 (4.9)
Divorced/separated/widowed	5 (3.0)	No. of living children^b^	
Missing	10 (6.1)	1	36 (27.7)
Religion		2–4	72 (55.4)
Orthodox	106 (64.2)	>4	22 (16.9)
Protestant	27 (16.4)	Mean (SD)	2.9 (1.9)
Muslim	22 (13.3)	Duration of IUD use, months	
Catholic	2 (1.2)	<6	32 (19.4)
Missing	8 (4.8)	6–12	18 (10.9)
Educational level		13–24	29 (17.6)
No education	61 (37.0)	25–36	23 (13.9)
Primary	47 (28.5)	37–60	22 (13.3)
Secondary	34 (20.6)	61–84	12 (7.3)
More than secondary	16 (9.7)	>84	10 (6.1)
Missing	7 (4.2)	Missing	19 (11.5)
Age		Mean (SD)	30.5 (31.3)
<20	9 (5.5)		
20–24	34 (20.6)		
25–29	49 (29.7)		
30–34	32 (19.4)		
>34	34 (20.6)		
Missing	7 (4.2)		
Mean (SD)	28.5 (6.7)		

Abbreviations: IUD, intrauterine device; SD, standard deviation; SNNPR, Southern Nations, Nationalities, and People’s Region.

a Denominator is 142 women who had previously been pregnant.

b Denominator is 130 women who had ever given birth.

Of these 165 clients discontinuing use of the IUD, 158 responded to our query about the reason for method discontinuation. As shown in Table 5, a plurality (43%) of the women discontinuing indicated they did so because of a desire to become pregnant. About one-quarter of the clients (25.9%) identified side effects or health concerns as an impetus for seeking removal services, and 20.3% indicated husband/partner disapproval as a reason for discontinuing use. A smaller proportion of women identified disapproval from another family member (7%), infrequent sex (5.7%), or method failure (2.5%) as reasons for discontinuation.

The most commonly cited reason for discontinuing use of the IUD was to become pregnant.

**TABLE 5:** Reasons for Seeking IUD Removal^a^ (N = 158)

	No. (%)
Want to become pregnant	68 (43.0)
Side effects/health concerns	41 (25.9)
Husband/partner disapproves	32 (20.3)
Other family member disapproves	11 (7.0)
Infrequent sex/husband away	9 (5.7)
Became pregnant while using	4 (2.5)
Don’t want to give a reason	4 (2.5)
Wants more effective method	3 (1.9)
Marital dissolution/separation	3 (1.9)
Completion of effective duration of use	2 (1.3)
I don’t think I can be pregnant/menopausal	1 (0.6)
Lack of access/too far for facility	1 (0.6)
Other^b^	7 (4.4)

a Women selected as many reasons as applied to their situation (categories are not mutually exclusive).

b Includes age, switched methods, and acquired a sexually transmitted infection.

### Facilitators to IUD Use

#### Satisfaction With the Method

For the most part, clients seeking both insertion and removal reported satisfaction with the IUD during the qualitative interviews. Clients cited the following facilitating factors to IUD uptake:

- The method is nonhormonal, long-acting, and highly effective.
- There is no need to return to the health center for method resupply (e.g., as with oral contraception and injectables).
- There is less room for human error leading to method failure.
- The IUD can be removed at any time.

#### Confidence Among Service Providers

Service providers, facility heads, and *woreda* heads reported feeling comfortable providing IUD services, and they noted that the immediate provision of resources and supplies following training, routine follow-up and mentorship, and performance review meetings contributed to their comfort with inserting and removing the IUD.

### Barriers to IUD Use

#### Sociocultural Barriers

Clients and service providers noted challenges posed by persistent myths and misconceptions about the method (i.e., the IUD will migrate throughout the body; the IUD causes infertility, uterine cancer, or hypertension). Limited community awareness of the method and husband/partner disapproval were also mentioned as barriers to uptake. Interestingly, respondents also noted that the long period of IUD efficacy (up to 12 years) sometimes deters clients. Despite the fact that the IUD can be removed at any point in time, clients reported uncertainty about adopting a method that is effective for such an extended period of time when they want to avoid pregnancy for only a few years.

#### Health System Barriers

Respondents also mentioned structural barriers to IUD uptake, including insufficient space in health centers to insert/remove IUDs, leading to privacy concerns, and discomfort with a male provider inserting the IUD (i.e., clients prefer a female provider but given that there are roughly twice the number of male providers than female providers, coupled with health worker shortages more broadly, this often is not feasible). While providers and clients noted the critical importance of counseling, both groups expressed concerns about the insufficient amount of time that is available for counseling given provider workloads. Respondents also noted that a substantial proportion of women do not return for follow-up appointments.

## DISCUSSION

During the 6-month study period (March–August 2014), 2,943 women sought IUDs at the 40 selected health centers in Ethiopia, constituting 5.7% of total contraceptive use. This represents a 14-fold increase from the 6-month period preceding IFHP+ support for the government’s IUD initiative (July–December 2011), when just 0.4% of clients seeking services in the 40 health centers accepted the method. This increase in IUD uptake is also reflected in the nationally representative 2014 mini Demographic and Health Survey (DHS). Data for this survey were collected by the Ethiopian Central Statistics Agency from January to April 2014 (overlapping with our study period), and revealed that IUDs constituted 1% of contraceptive use in Ethiopia. Considering that IUD use remained stagnant from 2005 to 2011 (0.2% and 0.3%, respectively), the increase to 1% within a 3-year period is notable.^8^^–^^10^ Knowledge of IUDs also improved over this time period, from 26.3% of women reporting familiarity with IUDs in 2011 to 37.8% in 2014.^9^^,^^10^ Findings from our study—as well as from the recent DHS—suggest that latent demand for the IUD is prevalent in Ethiopia. Evidence from Kenya, Madagascar, Nigeria, Tanzania, Uganda, and Zambia (and 7 other non-sub-Saharan African countries) also demonstrates substantial demand for IUDs when they become available.^5^

Our study corroborates previous findings that IUDs are acceptable to a wide range of women and adolescents,^5^ including new contraceptive users (18% of IUD adopters in our study were new users), those with little to no education (45% of the acceptors reported no educational attainment), those from rural areas (63% of acceptors), and younger users (59% were younger than 30, with 28% under the age of 25), and presumably as a means for both spacing and limiting pregnancies (given the relatively wide age and parity distribution of acceptors). Despite their acceptability, however, underlying bias often dissuades providers from offering the IUD as part of a comprehensive method mix—particularly to adolescents and nulliparous women.^12^^-^^14^

IUDs appear to be acceptable to a wide range of women, including young women.

In the 40 health centers participating in the study, introduction of IUDs into the method mix was correlated with a statistically significant reduction in reliance on short-acting methods (particularly injectables and oral contraceptives), from 93.1% to 79.5%, and increased reliance on all LARCs (both IUDs *as well as* implants), from 6.9% to 20.5%. Previous research has shown that the introduction of new contraceptive methods often results in a net benefit for *all modern* contraceptive methods.^15^ However, our findings show a sizable, statistically significant shift *toward* LARCs and away from short-acting methods (with the exception of condom use) following introduction of IUDs. Worth noting, IFHP+ has also invested heavily in expanding availability of contraceptive implants (Implanon) in the country.^16^ It is plausible that IFHP+’s simultaneous efforts to increase access to and uptake of both LARCs may have been mutually reinforcing, resulting in a concurrent increase in both methods.

Introduction of IUDs into the 40 health centers was correlated with a significant increase in the contribution of all LARCs to the method mix.

Our study (as well as the entirety of IFHP+’s support for the government’s IUD and implant initiatives) occurred in the public health sector. Given that a significant proportion of women in the region obtain contraception from the public sector, IFHP+’s experience represents an important contribution to the evidence base. Our study suggests that the IFHP+ model—composed of provider training, provision of post-training kits and consumables that enabled immediate initiation of service provision, routine provider follow-up and mentorship, and demand creation activities—is valid and can increase access to IUDs in a large public-sector program.

Due to the nature of our study design, we were unable to rigorously evaluate client follow-up; however, during FGDs and KIIs, respondents reported the perception that a substantial proportion of clients do not return for follow-up appointments. This could be attributable to lengthy distances to facilities, lack of money for transportation, and limited impetus to seek follow-up when the client experiences no problems.

Among the 165 clients seeking IUD removal services, 43% indicated that the decision to discontinue use was influenced by a desire to become pregnant. A quarter of clients (25.9%) identified health concerns/side effects as a reason for seeking removal, and 20.3% reported that husband/partner disapproval was a reason for discontinuation. The majority (61.8%) of those who discontinued had used the IUD for less than 36 months (and 30.3% for less than 1 year); however, our study design did not allow us to correlate reasons for discontinuation with duration of use for individual clients. Future research is needed to better understand the reasons for discontinuation by length of use.

Despite some debate about the cost-effectiveness of IUDs when they are used for less than their full efficacious period, recent research shows that if the IUD is used for a minimum of 2.1 years, the method is still more cost-effective than short-acting methods.^17^ The mean duration of use among clients seeking removal services during the study period was 2.6 years, and 52% of clients continued IUD use for at least 2 years.

According to a review of global access to and uptake of IUDs, favorable health policy and provider training are often predictive of increased IUD access; however, the specific facilitators and barriers to uptake are complex, country-specific, and poorly documented.^18^ The qualitative component of our study aimed to explore these specific facilitators and barriers in Ethiopia. Facilitators to use included the following: the method is nonhormonal, long-acting, and highly effective; there is no need to return to the health center for method resupply; there is less room for human error; and the IUD can be removed at any time. Similar to previous findings, our study found that barriers to IUD uptake included myths about the method (including misconceptions pertaining to cancer, infertility, and IUD migration), limited community awareness, partner disapproval, and infrastructure deficits at health facilities.^13^^,^^19^ While provider bias is a common barrier to provision of IUDs, providers participating in our study reported that they felt comfortable inserting and removing the method—possibly attributable to IFHP+’s rigorous competency-based trainings. Finally, in settings such as Ethiopia, it is particularly important that clients are fully informed that they can have the IUD removed at any time, given that the extended period of IUD effectiveness was highlighted as a deterrent to use during FGDs.

Women liked the IUD because it is nonhormonal, long-acting, and highly effective, among other reasons.

While acceptability of the IUD is growing, limited community awareness, myths and misconceptions about the IUD, and infrastructure deficits at health centers must be addressed to further expand availability of a comprehensive method mix. Our findings point to the need for intensified awareness creation for client cohorts and their partners (especially given that just 17.1% of women reported hearing about the IUD from a health extension worker) and mechanisms for ensuring that counseling is improved at the facility level. While health extension workers did include discussion on myths and misconceptions about the IUD in their sensitization efforts at the community level, we would recommend that more intentional, tailored messaging around specific myths and misconceptions be developed for future interventions.

While acceptability of the IUD is growing, barriers such as myths and misconceptions and infrastructure deficits must be addressed to further expand availability of a broad range of contraceptive methods.

### Limitations

Our study had several limitations. Because of the study’s cross-sectional design, we were able to collect data from clients only at one point in time and, as such, were unable to assess follow-up, client satisfaction, and potential side effects. Additionally, because IUDs were just becoming available during the study period, our sample size for clients seeking IUD removal services (n = 165) was quite small. Furthermore, we obtained data pertaining to the contraceptive method mix before and after introduction of IUDs from family planning registers within a purposively selected sample of 40 health facilities. As the selection was not random, the results presented may overestimate the increase in LARC use. The study team aimed to ensure selection of sites that provided IUD services and had high client volume; thus, these conditions may not be representative of all health facilities supported by IFHP+. In addition, while we have taken great care to ensure accurate review and management of health facility register data during data extraction, we are cognizant that there is potential for data quality issues when relying on a retrospective review of health facility data. Social desirability bias may also have been operative due to the sensitive nature of the questions asked in both the cross-sectional survey and qualitative interviews. Contraception can be a sensitive issue within conservative communities; therefore, participants may answer a question in a way that will be viewed more favorably by the research team. To limit this bias, the survey questionnaires and qualitative interview guides were pilot tested in the community prior to implementation. Finally, the cross-sectional survey and qualitative interviews were implemented among women obtaining IUD services in a health facility; therefore, there is potential for selection bias as women who come to the health facilities are likely to have better health-seeking behavior and access to health information than their peers. Thus, the results of this study may not be generalizable to Ethiopian women more broadly.

## CONCLUSION

Through provider training, provision of post-training supplies, and community-based awareness creation strategies, it is possible to expand access to IUD services and thereby to increase method choice and use of IUDs, as demonstrated in our study conducted at the public health facility level in Ethiopia. During the 6-month study period, we found the IUD to be acceptable to a diverse range of Ethiopian women, including those from rural areas, those with little to no education, and younger women. Echoing programmatic experience across sub-Saharan Africa,^6^^,^^7^ our study suggests that unmet need for the IUD may be more prevalent in Ethiopia than previously thought. Given that IUDs constitute such a small proportion of contraceptive use throughout sub-Saharan Africa, our study presents relevant considerations for ensuring that clients’ contraceptive choice is respected and fulfilled across the region.

## Notes

**Peer Reviewed**
